# The efficacy and adverse events of delafloxacin in the treatment of acute bacterial infections: A systematic review and meta-analysis of randomized controlled trials

**DOI:** 10.3389/fphar.2022.975578

**Published:** 2022-09-28

**Authors:** Rong He, Fei Lin, Bin Yu, Jingyue Qiu, Lingli Zheng

**Affiliations:** ^1^ Department of Respiratory and Critical Care Medicine, The First Affiliated Hospital of Chengdu Medical College, Chengdu, China; ^2^ Department of Pharmacy, The First Affiliated Hospital of Chengdu Medical College, Chengdu, China; ^3^ Sichuan Province College Key Laboratory of Structure-Specific Small Molecule Drugs, Chengdu Medical College, Chengdu, China; ^4^ Department of Pharmacy, Mianyang Central Hospital, Mianyang, China; ^5^ Department of Pharmacy, PLA Strategic Support Force Medical Center, Beijing, China

**Keywords:** delafloxacin, acute bacterial infections, S. aureu, meta analysis, efficacy

## Abstract

**Background:** This study aims to assess the clinical efficacy and adverse events of delafloxacin for the treatment of acute bacterial infections in adult patients through meta-analysis.

**Methods:** The PubMed, Embase, Cochrane library, Web of Science, and Clinical trails databases were searched up to 26 March 2022. Only randomized controlled trials (RCTs) that evaluated delafloxacin and comparator antibiotics for treating acute bacterial infections in adult patients were included. The clinical cure rate and microbiological eradication rate at the posttreatment evaluation, while the secondary outcomes included the risk of adverse events (AEs).

**Results:** In total, six randomized controlled trials (RCTs) involving 3,019 patients with acute bacterial infection were included. There were no significant differences in the clinical cure rate between delafloxacin and comparators (OR = 1.06%, 95% CI = 0.89–1.26, I^2^ = 0%). Overall, the results showed that delafloxacin had a microbiological eradication rate (documented and presumed) similar to the comparators (OR = 1.33%, 95% CI = 0.94–1.88, I^2^ = 0%) in the pooled analysis of the six studies. Any treatment-emergent adverse events (TEAEs) did not show significant differences between delafloxacin and the comparators (OR = 0.93%, 95% CI = 0.80–1.08, I^2^ = 75%). Serious adverse events (SAEs) did not differ between the delafloxacin and comparators (OR = 0.94%, 95% CI = 0.67–1.32, I^2^ = 0%). The results of gastrointestinal disorders were (OR = 1.26%, 95% CI = 1.01–1.56, I^2^ = 89%), and nausea, vomiting, and diarrhea were (OR = 0.77%, 95% CI = 0.45–1.34, I^2^ = 79%), (OR = 1.00%, 95% CI = 0.74–1.36, I^2^ = 72%), and (OR = 2.10%, 95% CI = 1.70–2.96, I^2^ = 0%), respectively. The results showed that there was no significant difference in the incidence of nausea and vomiting between delafloxacin and the comparator, but the incidence of diarrhea was higher. The analysis of neurological disorders indicated that the incidence of nervous system disorders was lower in the delafloxacin group (OR = 0.71%, 95% CI = 0.50–1.01, I^2^ = 52%).

**Conclusion:** The clinical efficacy, microbiological eradication rate and the incidence of AEs of delafloxacin in the treatment of acute bacterial infections were similar to those of the comparators, as an alternative therapeutic agent.

## Introduction

Delafloxacin is a novel anionic fluoroquinolone antibiotic that is administered as a broad-spectrum antibacterial drug, approved by the United States Food and Drug Administration (FDA) in June 2017, for the treatment of acute bacterial skin and skin structure infection (ABSSSI) (Markham, 2017). The bactericidal action of delafloxacin results from dual inhibition of topoisomerase II and IV, which are required for bacterial DNA replication, transcription, repair, and recombination. Delafloxacin has an anionic structure, which influx of the drug into bacterial cells in an acidic environment; infection settings have an acidic environment (Shiu et al., 2019). After the molecule enters the cell at neutral pH, delafloxacin deprotonates and remains in the bacteria as an ion (Shiu et al., 2019). This property is unique compared to other fluoroquinolones, such as moxifloxacin. The minimum inhibitory concentration (MIC) of moxifloxacin, ciprofloxacin, levofloxacin, and other quinolones increases with the decrease in pH, but the antibacterial activity decreases. The unique anionic properties of delafloxacin and its potency in acidic environments, make delafloxacin several times more potent than levofloxacin (64-fold MIC) (Correia et al., 2017; Scott, 2020). Therefore, compared to other fluoroquinolones, delafloxacin has strong activity against a large number of drug-resistant bacteria including Gram-positive bacteria (such as *Staphylococcus aureus*, *Enterococcus faecalis*, and *Enterococcus faecium*), Gram-negative bacteria (such as *Klebsiella pneumoniae*, *Escherichia coli*, *Pseudomonas aeruginosa*, and *Acinetobacter baumannii)*, atypical bacteria (such as *Chlamydia pneumonia*, *Mycoplasma pneumonia* and *Legionella pneumonia*), and anaerobic bacteria. It can also be used to treat various of acute infectious diseases (Jorgensen et al., 2018; McCurdy et al., 2020; Scott, 2020). Owing to its good pharmacokinetic properties, broad-spectrum, and sequential therapeutic potential (Shiu et al., 2019). Cross-resistance was found between delafloxacin and other quinolones *in vitro*. However, some quinolone-resistant clinical isolates might still be sensitive to delafloxacin. The *in vitro* activity of delafloxacin against methicillin-resistant *Staphylococcus aureus* (MRSA), methicillin-susceptible *S. aureus* (MSSA), *E. faecalis*, *E. faecium*, *E. coli* and *K. pneumoniae* isolates was found to be (MIC_90_) 0.5 μg/ml, 0.008 μg/ml, 1 μg/Ml, >4 μg/ml, >4 μg/ml, and >4 μg/ml, respectively (Kocsis et al., 2021). The delafloxacin MIC_90_ value against levofloxacin-non-susceptible *S. aureus*, and MRSA isolates was 0.25 μg/ml. Delafloxacin has a lowly toxicity of quinolones derived from the toxicity of quinolones, and can be used for intravenous and oral administration (Bush et al., 2020).

Some studies have suggested that delafloxacin has better clinical efficacy and safety than other antibiotics (Bassetti et al., 2019; Giordano et al., 2019). However, other studies have shown no significant difference in the efficacy of delafloxacin compared to that of conventional antibiotics and a higher incidence of some adverse events (Lan et al., 2019; Shiu et al., 2019). In recent years, with the emergence of drug-resistant bacteria and an increase in the indication of delafloxacin in acute bacterial infections, it is necessary to systematically evaluate the clinical efficacy and safety of delafloxacin in the treatment of acute bacterial infections.

In this study, delafloxacin, the latest generation of broad-spectrum fluoroquinolone antibacterial drug, was investigated, and its clinical efficacy and safety were compared in the treatment of acute bacterial infections to provide a reference for clinical application.

## Materials and methods

### Study search and selection

We searched PubMed, Embase, Cochrane Library, Web of Science, and Clinical Trial from inception to 26 March 2022. The search terms used were “delafloxacin” OR “ABT-492” OR “RX-3341-83.” The studies were included if they met the following criteria: 1) RCT, 2) patients diagnosed with acute bacterial infection; 3) age ≥18 years; 4) intervention of delafloxacin, and comparison with other antibiotics; 5) outcome of efficacy, including clinical and microbiological response, adverse events (AEs). Two investigators (Yu and Qiu) independently screened and reviewed each study. Any disagreement was resolved by consulting a third reviewer (Lin). Studies published only in English were included. Data were extracted independently by two researchers (He and Zheng). In case of disagreements during data extraction, the issue was checked and resolved by the third researcher (Lin). The data extracted from the included studies were on the authorship, year of publication, study design, study duration, study site, study population, antibiotic regimens of delafloxacin and the comparators, clinical and microbiological outcomes, and risk of AEs. Ethical approval was not necessary for meta-analysis in our institute.

### Outcome measurement

The primary outcome was overall clinical cure with complete or near complete resolution of baseline signs and symptoms of the primary infection with no further need for antibacterial treatment, which was evaluated by the investigator during follow-ups. The microbiological response and AEs were evaluated as secondary outcomes. The microbiological response was defined as microbiological eradication or presumed eradication. AEs were recorded, respective of causality.

### Data analysis

The overall quality of evidence was evaluated using GRADE (Grading of Recommendations, Assessment, Development, and Evaluation). The summary of the risk of bias for each included RCT was assessed using the Cochrane risk-of-bias tool (Higgins et al., 2011). Two reviewers subjectively reviewed all included studies and rated them “low risk,” “high risk,” or “unclear risk” according to the items of the tool. All statistical analyses were performed using Review Manager version 5.3. Pooled odds ratios (ORs) and 95% confidence intervals (CIs) were used as the measures of association between outcomes and the use of delafloxacin. Study heterogeneity was presented using the Chi-squared based on Cochran’s Q statistic and I^2^. The heterogeneity was considered to be significant at *p* < 0.10% or I^2^ >50%. The fixed-effect model was used when data were homogenous, and the random-effect model and sensitivity analysis were used when data were significantly heterogeneous. A sensitivity analysis was conducted using the leave-one-out approach.

## Results

### Search and study characteristics

**FIGURE 1:**
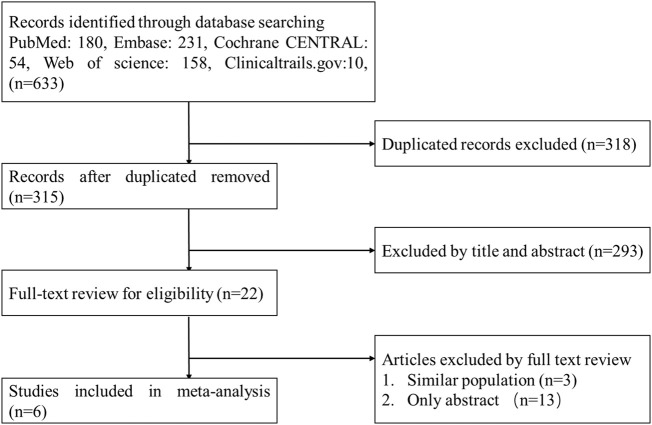
Flowchart of the study selection process.

**FIGURE 2:**
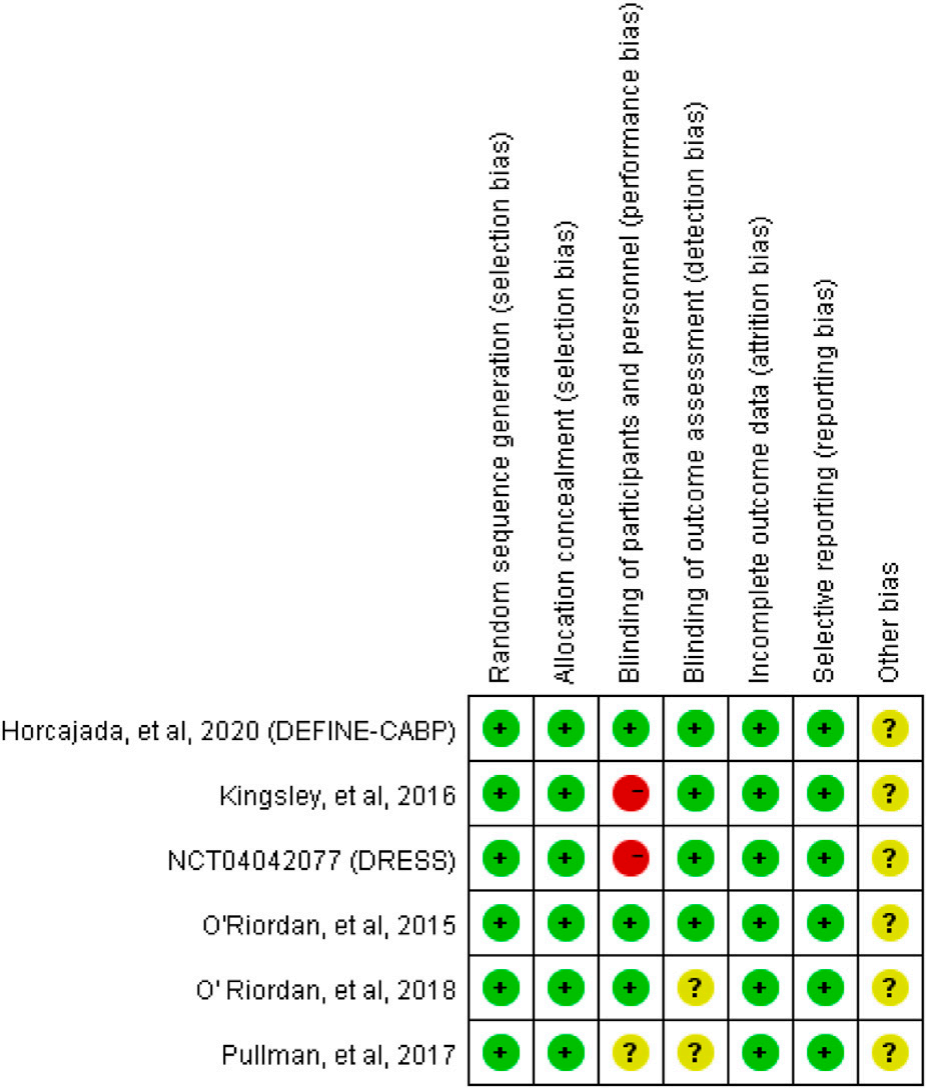
Risk of bias summary.

**FIGURE 3:**
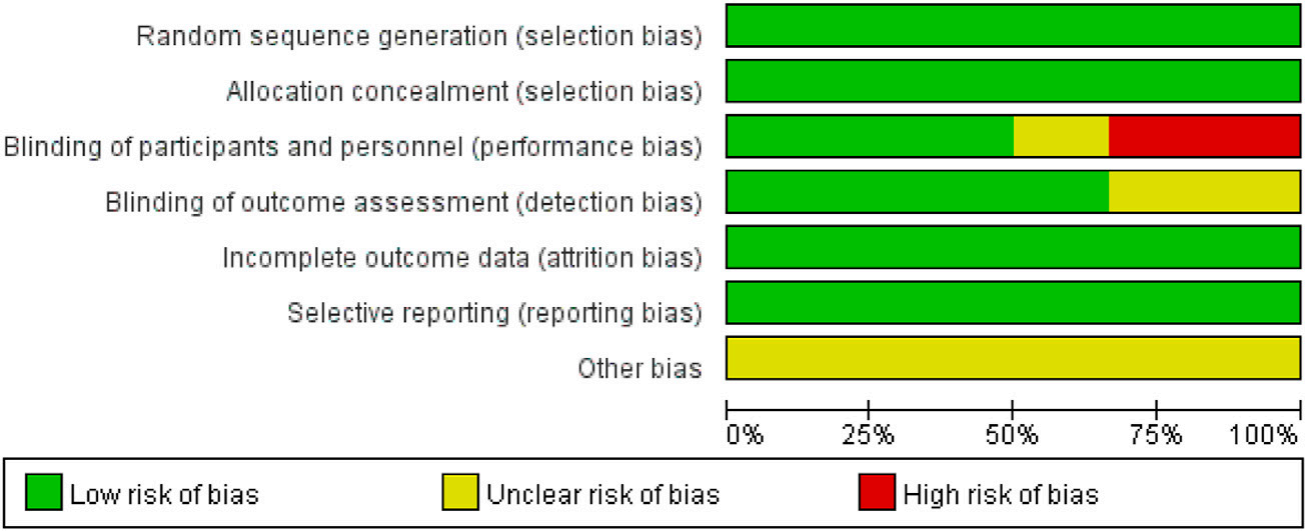
Risk of bias graph.

A flow diagram of the study selection is presented in Figure 1. The search program yielded 633 references, from PubMed (N = 180), Embase (N = 231), Cochrane Library (N = 54), Web of Science (N = 158), and Clinical Trails (N = 10), After excluding 318 duplicates, the remaining 315 abstracts were screened. In total, six studies were included with 2,990 participants in the systematic review and meta-analysis. Of these, five studies were published in full text. One additional eligible study (NCT04042077) that was completed but not published was retrieved from Clinical Trials. Six RCTs published between 2015 and 2020, met the inclusion criteria. Five studies were multicenter, double-blind, intention-to-treat analyses, and an RCTs, and one study was multicenter, observer-blinded, intention-to-treat analyses, and RCT (Table 1). The experimental groups treated with delafloxacin and other antibiotics consisted of 1,449 and 1,541 patients, respectively (Table 2). The baseline demographic characteristics were not balanced in the pooled delafloxacin and comparator groups (Table 3). Overall, males accounted for 60.2% and 62.0% of the population in the delafloxacin and other antibiotic groups, respectively (Table 3). In the delafloxacin and other antibiotic groups, patients were 53.3 ± 17.00 and 51.9 ± 16.94 years old, respectively (Table 3). Caucasians accounted for 88.2% and 87.7% of the population in the delafloxacin and other antibiotic groups, respectively (Table 3). Two studies were conducted in only the United States, and the other four studies were conducted in multiple countries. O’Riordan (O'Riordan et al., 2015) compared two doses of delafloxacin (300 mg intravenous and 450 mg intravenous) and comparators, but only included the data of 300 mg; the other five studies used delafloxacin (300 mg; intravenous) in the experimental group. The patients received delafloxacin (300 mg; intravenous) every 12 h for 3 days, and the treatment was switched to 450 mg oral delafloxacin in another study by O’ Riordan (O'Riordan et al., 2018). In a study by Horcajada (Horcajada et al., 2020), patients received delafloxacin (300 mg) every 12 h intravenously at least 6 times, and the treatment was switched to an oral administration of 450 mg of delafloxacin. The total duration of treatment (intravenous and oral) was 5–10 days. All patients diagnosed with ABSSSIs and cSSSI received 300 mg of intravenous every 12 h for 5–14 days (based on the investigator’s judgment) (O'Riordan et al., 2015; Kingsley et al., 2016; Pullman et al., 2017). The patients in the NCT04042077 study received 300 mg of intravenous delafloxacin every 12 h, and the treatment was switched to 450 mg of oral delafloxacin for 5–14 days (based on the investigator’s judgment). Two studies used vancomycin plus aztreonam as comparators, one study used tigecycline, one study used linezolid or vancomycin, and one study used the best available therapy, including vancomycin, linezolid, piperacillin/tazobactam or tigecycline. Overall, 1,449 and 1,541 patients comprised the delafloxacin group and the comparator group, respectively. Five studies were focused on tissue and skin infection diseases. In total, 1,827 male patients and 1,214 female patients were included in the study. The risk of bias in the included studies is presented in Figures 2, 3; only two studies had a high risk of bias concerning the blinding of participants and performance.

**TABLE 1:** Characteristics of six studies.

Study, year	Study duration	Study site	Study population	Number of patients		Dose regimen		Therapy duration
				Delafloxacin	Comparator	Delafloxacin	Comparator	
O'Riordan et al. (2015)	Between June and September 2008	14 sites in United States	>18 years, cSSSI	49	50	300 mg q12 h	tigecycline 100 mg IV once, followed by 50 mg IV q12 h	5–14 days
Kingsley et al. (2016)	Between February and November 2011	23 sites in United States	18 years, ABSSSI	81	Vancomycin (n = 98), Linezolid (*n* = 77)	300 mg q12 h	15 mg/kg vancomycin, 600 mg linezolid	5–14 days
Pullman et al. (2017)	Between April 2013 and June 2014	34 sites in 7 countries	18 years, ABSSSI	331	329	300 mg q12 h	vancomycin 15 mg/kg plus aztreonam 2 g q12 h	5–14 days
O'Riordan et al. (2015)	Between May 2014 and January 2016	76 sites in 16 countries	18 years, ABSSSI	423	427	300 mg q12 h or 450 mg BID	vancomycin 15 mg/kg plus aztreonam 2 g q12 h	5–14 days
Horcajada et al. (2020)	Between December 2016 and August 2018	88 sites in 18 countries	≥18 years, CAP	431	428	300 mg q12 h	moxifloxacin 400 mg q24 h (MRSA linezolid 600 mg IV q12 h)	5–10 days
NCT04042077	Between September 2019 and October 2020	12 countries	≥18 Years, SSIs	134	Vancomycin (2) Linezolid (8) Piperacillin/Tazobactam (68) Tigecycline (54)	300 mg q12 h or 450 mg BID	Vancomycin 15 mg/kg BID Linezolid 600 mg BID Piperacillin/Tazobactam 4.5 g TID Tigecycline 50 mg TID	5–14 days

cSSSI, complicated skin and skin-structure infections; ABSSSI, acute bacterial skin and skin structure infections; CAP, community-acquired bacterial; SSIs, Surgical Site Infections.

**TABLE 2:** Characteristics of enrolled patients.

Study	Population	ITT		mITT		CE		ME	
		Delafloxacin	Comparator	Delafloxacin	Comparator	Delafloxacin	Comparator	Delafloxacin	Comparator
O'Riordan et al. (2015)	99	49	50	99	50	75	34	59	24
Kingsley et al. (2016)	256	81	175	51	124	60	131	34	91
Pullman et al. (2017)	660	331	329	243	247	294	297	220	225
O' Riordan et al. (2018)	850	423	427	275	277	395	387	264	250
Horcajada et al. (2020)	859	431	428	257	263	394	389	240	248
NCT04042077	266	134	132	105	102	128	127	94	81

ITT, intent-to treat; mITT, microbiological intent-to treat; CE, clinically evaluable; ME, microbiological evaluable.

**TABLE 3:** Baseline demographic characteristics of the study populations.

	Male (%)		Mean ± SD. age (years)		Race, n (%) of delafloxacin				Race, n (%) of comparator			
	Delafloxacin	Comparator	Delafloxacin	Comparator	Caucasian	Black	Asian	Other	Caucasian	Black	Asian	Other
O'Riordan et al. (2015)	31 (63.3)	35 (70.0)	42.7 ± 15.10	40.4± 13.83	39 (79.6)	7 (14.3)	0	3 (6.1)	40 (80.0)	9 (18.0)	0	1 (2.0)
Kingsley et al. (2016)	49 (60.5)	103 (58.9)	39.7 ± 14.26	44.8± 15.22	63 (77.8)	10 (12.3)	0	8 (9.9)	132 (75.4)	30 (17.1)	0	13 (7.4)
Pullman et al. (2017)	206 (62.2)	209 (63.5)	46.3 ± 13.91	45.3 ± 14.4	297 (89.7)	27 (8.2)	1 (0.3)	6 (1.8)	304 (92.4)	19 (5.8)	1 (0.3)	4 (0.1)
O' Riordan et al. (2018)	262 (61.9)	276 (64.6)	51.2 ± 15.98	50.2± 16.03	348 (82.3)	13 (3.1)	11 (2.6)	51 (12.1)	355 (83.1)	18 (4.2)	15 (3.5)	39 (9.1)
Horcajada et al. (2020)	251 (58.2)	253 (59.1)	60.7 ± 16.06	59.3 ±16.58	398 (92.3)	22 (5.1)	5 (1.2)	6 (1.4)	388 (90.7)	33 (7.7)	5 (1.2)	2 (0.5)
NCT04042077	73 (54.5)	79 (59.8)	66.0 ± 13.65	63.7 ±13.71	133 (99.7)	1 (0.7)	0	0	132 (100.0)	0	0	0
Total	872 (60.2)	955 (62.0)	53.3 ± 17.00	51.9 ± 16.94	1278 (88.2)	80 (5.5)	17 (1.2)	74 (5.1)	1351 (87.7)	109 (7.1)	21 (1.4)	59 (3.8)

The assessment of quality using GRADE criteria found high-quality evidence in all analyses performed as a result of a large number of participants and blinding in most studies (Supplementary Table S1).

### Clinical response

**FIGURE 4:**
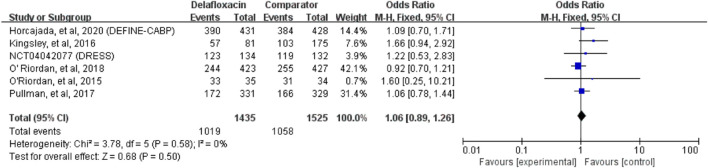
Overall clinical cure rates of delafloxacin and comparators in the treatment of acute bacterial infections.

Overall, the clinical cure ratio of delafloxacin was similar to that of the comparators in the treatment of acute bacterial infections (OR = 1.06%, 95% CI = 0.89%–1.26%, I^2^ = 0%, Figure 4) in the pooled analysis of 6 studies. No significant difference was found between delafloxacin and vancomycin in the pooled analysis of 3 studies (OR = 0.98%, 95% CI = 0.79%–1.20%, I^2^ = 51%).

### Microbiological response

**FIGURE 5:**
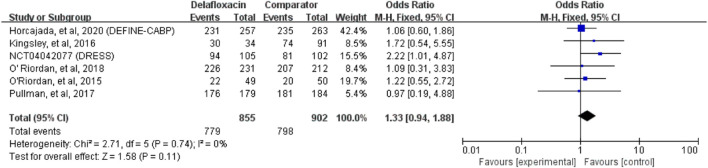
Overall microbiological eradication rates of delafloxacin and comparators in the treatment of acute bacterial infections.

**FIGURE 6:**
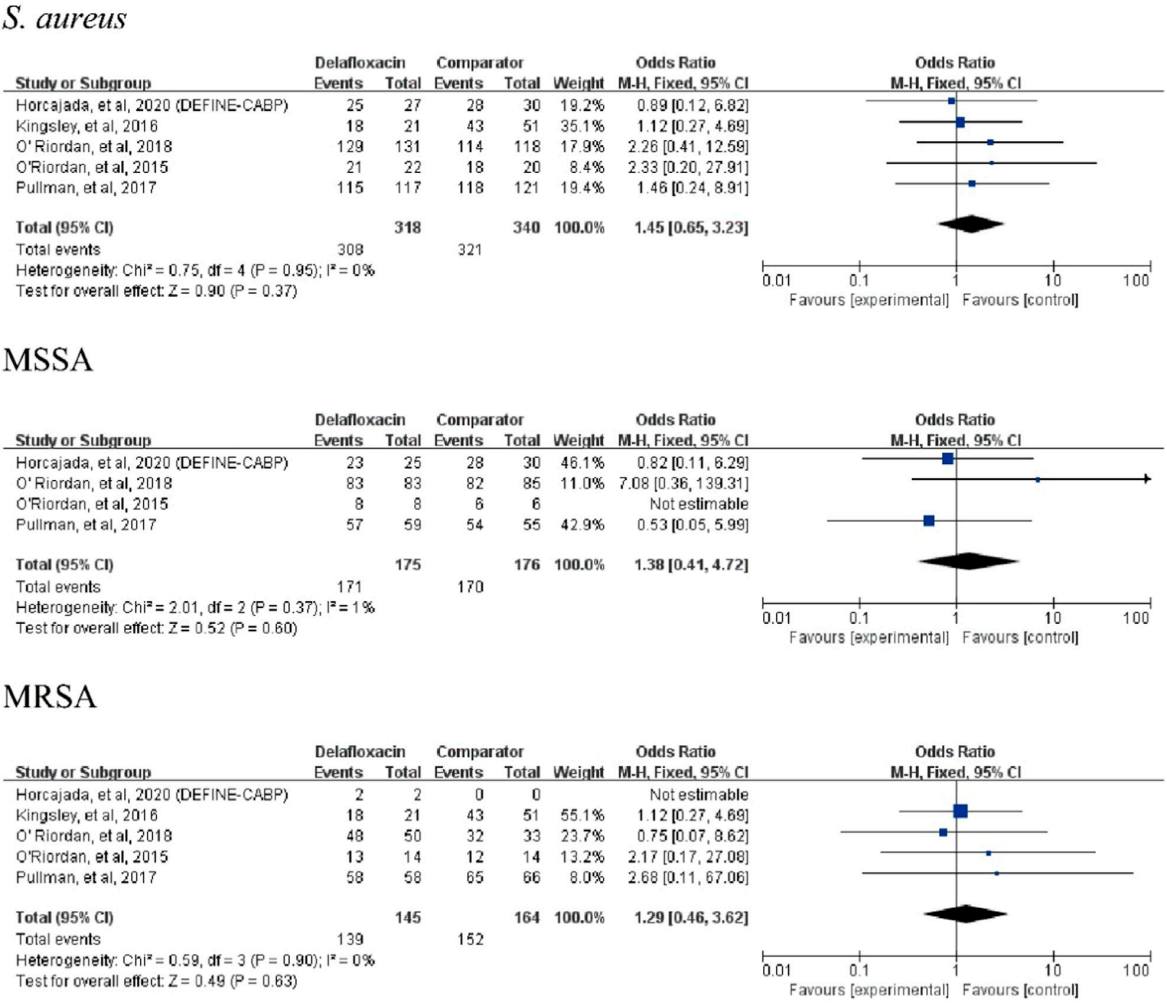
Overall *S. aureus* eradication rates of delafloxacin and comparators in the treatment of acute bacterial infections.

Delafloxacin had a microbiological eradication rate (documented and presumed) similar to that of the comparators in the treatment of acute bacterial infections (OR = 1.33%, 95% CI = 0.94%–1.88%, I^2^ = 0%) in the pooled analysis of the six studies (Figure 5). Five studies reported objective responder rates among microbiologically evaluated populations; no significant differences were found between delafloxacin and comparators regarding the infection caused by *S. aureus* (OR = 1.45; 95% CI = 0.65–3.23, I^2^ = 0%), MRSA (OR = 1.29, 95% CI = 0.46–3.62, I^2^ = 0%), and methicillin-susceptible *S. aureus* (MSSA; OR = 1.38, 95% CI = 0.41–4.72, I^2^ = 1%) (Figure 6).

### Adverse events

**FIGURE 7:**
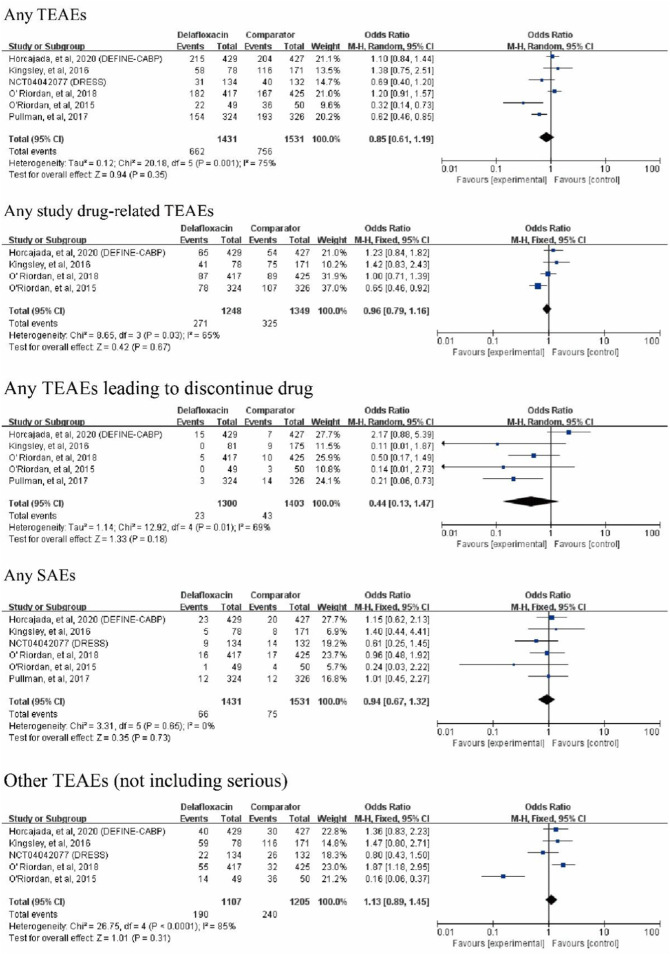
Risk of adverse events between delafloxacin and comparators in the treatment of acute bacterial infections.

Any treatment-emergent adverse events (TEAEs) did not differ between delafloxacin and comparators (OR = 0.93%, 95% CI = 0.80%–1.08%, I^2^ = 75%). Serious adverse events (SAEs) did not differ between delafloxacin and the comparators (OR = 0.94%, 95% CI = 0.67%–1.32%, I^2^ = 0%). The other adverse event did not differ between delafloxacin and the comparator (OR = 1.13%, 95% CI = 0.89–1.45, I^2^ = 85%). The risks of TEAEs related to the studied drug were similar between delafloxacin and the comparators (OR = 0.96; 95% CI = 0.79%–1.16%, I^2^ = 65%). Finally, the risk of discontinuation of the study drug due to AEs was lower for delafloxacin than for the comparators (OR = 0.59%; 95% CI = 0.36%–0.99%, I^2^ = 69%, Figure 7). In the sensitivity analysis, after removing the data taken from the study by O ‘Riordan (O’Riordan et al., 2015), the heterogeneity of any studied drug-related TEAEs and other TEAEs (not including serious) decreased from 65% to 0% and 85%–35%, respectively. In the pooled analysis of four RCTs, all-cause mortality did not differ between the delafloxacin and the comparator (OR = 1.26%, 95% CI = 0.58%–2.75%, I^2^ = 0%).

A significant difference was found between delafloxacin and the comparators for the risk of gastrointestinal disorders (OR = 1.26%, 95% CI = 1.01%–1.56%, I^2^ = 89%). In the subgroup analysis of the different types of gastrointestinal disorders, including vomiting, nausea, and diarrhea, no significant difference was found between delafloxacin and comparators for the risk of vomiting (OR = 0.77%, 95% CI = 0.45%–1.34%, I^2^ = 79%) and nausea (OR = 1.00%, 95% CI = 0.74%–1.36%, I^2^ = 72%), however, a significant difference was found between delafloxacin and comparators for the risk of diarrhea (OR = 2.10%, 95% CI = 1.50%–2.96%, I^2^ = 0%). In the sensitivity analysis, after removing the data taken from the study by O ‘Riordan (O'Riordan et al., 2015), the heterogeneity of gastrointestinal adverse reactions, nausea and vomiting decreased from 89% to 0% (*p* < 0.0001), 72%–3% (*p* = 0.24), and 79%–0% (*p* = 0.16), respectively. Additionally, the study by O’Riordan et al. (2018) and the NCT04042077 study reported that 0.2% and 0.75% of patients in the delafloxacin group had *Clostridium difficile* diarrhea, respectively. The study by Horcajada et al. (2020) reported that 0.5% of patients in the delafloxacin group and 0.2% of patient in the moxifloxacin group had *Clostridium difficile* diarrhea.

The risk of nervous system disorders was not significantly different between delafloxacin and the comparators (OR = 0.71%, 95% CI = 0.50%–1.01%, I^2^ = 52%). But delafloxacin had a low risk of headache (OR = 0.63, 95% CI = 0.43–0.93, I^2^ = 0%). In the sensitivity analysis, after removing the data taken from the study by Kingsley (Kingsley et al., 2016), the heterogeneity of nervous system disorders decreased from 52% to 0% (*p* = 0.004).

## Discussion

Today, quinolones have been found over half a century from 1962. They have been widely used for over 30 years in the clinic for treating infectious diseases (Bush et al., 2020). In recent years, many quinolone-resistant bacteria have been isolated in hospitals (Correia et al., 2017). Delafloxacin is a new generation of broad-spectrum fluoroquinolone antibacterial agent approved by the FDA for treating skin and soft tissue infections (Markham, 2017). However, it has also been used to treat other acute infections due to the emergence of drug-resistant bacteria. The meta-analysis of safety and efficacy data from the 6 RCTs for patients with acute bacterial infection showed that there was no significant difference in the clinical efficacy of delafloxacin compared to that of the comparators in the treatment of adult patients. First, the clinical cure rate of delafloxacin in treating acute bacterial infection was as high as that of the comparators in the pooled populations of the 6 RCTs. Second, the microbiological eradication rate of delafloxacin was not significantly different from that of the comparators in the pooled analysis of the 6 RCTs. The results were similar to those found by Lan (Lan et al., 2019) for acute bacterial skin and skin structure infections. The microbiological eradication rate among the microbiologically evaluated population was not significantly different between delafloxacin and the comparators for *S. aureus*, MRSA, and MSSA. Our results were supported by the findings of Pullman (McCurdy et al., 2017) and O′ Riordan (O'Riordan et al., 2018) based on the MIC test, which showed that the MIC_50/90_ of delafloxacin values was 0.008/0.25 μg/ml and 0.12/0.25 μg/ml against *S. aureus* and MRSA isolates *in vitro*, respectively. McCurdy (McCurdy et al., 2020) found that the microbiological success rates were 92.6% for *S. aureus* (100% for MRSA). Like other quinolones, delafloxacin is available in intravenous and oral forms, which facilitates treatment in the outpatient setting, and has activity against Gram-positive and Gram-negative bacteria. However, linezolid and vancomycin only inhibit Gram-positive bacteria. Tigecycline is the last line of defense for the treatment of bacterial infections and is only administered intravenously (Yaghoubi et al., 2021). Overall, delafloxacin can be used for treating adult patients with acute bacterial infections or resistant bacterial infections, such as MRSA.

As for its safety, the risk of AEs is another important concern in the treatment of acute bacterial infections with delafloxacin. All included studies reported AEs after receiving delafloxacin and the comparator to varying degrees. In this analysis, the pooled risks of any TEAEs were similar between delafloxacin and comparators. The risk of TEAEs due to the investigated drugs, leading to discontinuation of treatment and SAEs did not differ significantly between delafloxacin and the comparators. The overall incidence of SAEs was lower than that in the study by Horcajada (Horcajada et al., 2020) and Kingsley (Kingsley et al., 2016). Moreover, delafloxacin was associated with a lower risk of discontinuation of the investigated drug due to AEs than the comparators. Similar results were found in the studies by Lodise (Lodise et al., 2018) and Lan (Lan et al., 2019) for acute bacterial skin and skin structure infections.

A comparison of general adverse reactions, high heterogeneity due to the small sample size, and results of sensitivity analysis revealed a significant difference between delafloxacin and the comparators. The most common AEs are nausea, diarrhea, vomiting, and headaches. The results of the meta-analysis showed that delafloxacin had a high incidence of gastrointestinal disorders and a low incidence of nervous system disorders. However, more studies to clarify this issue are needed in the future.

Additionally, some warnings regarding AEs are mentioned on delafloxacin product labels based on clinical experience and safety data from delafloxacin clinical trials, including tendinitis and tendon rupture (related to Achilles tendon, hand, biceps, thumb, and other tendons), peripheral neuropathy, central nervous system effects (hallucinations, anxiety, depression, insomnia, severe headache, and confusion), exacerbation of myasthenia gravis, hypersensitivity reactions, *Clostridium difficile*-associated diarrhea and bacterial drug resistance (Markham, 2017; Tulkens et al., 2019). Some studies have shown that it has no significantly affected on liver and kidney functions (Kocsis et al., 2021; O'Riordan et al., 2018). But, delafloxacin can also cause hypertransaminasemia. The risk of hypertransaminasemia was found to be 3.0% for patients in the delafloxacin group (Horcajada et al., 2020). Thus, hypertransaminasemia is mentioned among AEs by the FDA and European Medicines Agency (EMA).

This meta-analysis had one major strength. Six RCTs and three types of acute bacterial infections, ABSSSI, SSI and CABP were included in this meta-analysis. However, our study has some limitations. First, we only included RCTs and compared the effectiveness of the tested drug to that of other antibiotics to avoid possible confounding factors; we did not incorporate observational studies. Other biases might have been introduced by the RCT (e.g., non-representativeness of the population). Most participants were young and of Caucasian origin, and this might have affected the generalizability of the results. Second, data on the patients with severe infections, comorbidities, or under medication were excluded. This might have caused the results to show good efficacy of treatment with delafloxacin compared to the efficacy of the treatment of patients in the real world. Third, the lack of the median duration of intravenous and oral treatment in the studies might be an important issue in the use of novel antibiotics. Additionally, the efficacy of delafloxacin was assessed using an IV treatment in all the studies, but oral treatment should be administered whenever possible in clinical settings. Finally, delafloxacin has been tested on mild pneumonia patients, while other fluoroquinolones have been reserved for severe diseases. Therefore, more data needs to be analyzed after these trials are completed.

In conclusion, the clinical and microbiological efficacy of delafloxacin is as high as the comparators in the treatment of acute bacterial infection, and this antibiotic is as well-tolerated as the comparators. The results were consistent in the sensitivity analyses. Therefore, delafloxacin can be recommended as an appropriate antibiotic for treating acute bacterial infectious diseases.

## Data Availability

The original contributions presented in the study are included in the article/Supplementary Material, further inquiries can be directed to the corresponding authors.
