# Supplementary material for: The efficacy and adverse events of delafloxacin in the treatment of acute bacterial infections: A systematic review and meta-analysis of randomized controlled trials
Source: Front Pharmacol. 2022 Sep 28;13:975578. doi: 10.3389/fphar.2022.975578 (PMC9554268; doi:10.3389/fphar.2022.975578)
Supplement: Supplementary file 1 [file DataSheet1.docx]

Search strategy

Pubmed: "delafloxacin" [Supplementary Concept] OR ‘ABT-492’ OR ‘RX-3341-83’

Embase: 'delafloxacin'/exp OR delafloxacin OR 'abt 492'/exp OR 'abt 492' OR 'rx 3341 83'/exp OR 'rx 3341 83'

Cochrane Library: delafloxacin OR ABT-492 OR RX-3341-83

Web of Science: delafloxacin OR ABT-492 OR RX-3341-83

Clinical Trials: delafloxacin

**Supplemental Table S1.** GRADE risk of bias assessment

| **Certainty assessment** | | | | | | | **№ of patients** | | **Effect** | | **Certainty** | **Importance** |
| --- | --- | --- | --- | --- | --- | --- | --- | --- | --- | --- | --- | --- |
| **№ of studies** | **Study design** | **Risk of bias** | **Inconsistency** | **Indirectness** | **Imprecision** | **Other considerations** | **response** | **placebo** | **Relative (95% CI)** | **Absolute (95% CI)** |  |  |
| **cure ration** | | | | | | | | | | | | |
| 6 | randomised trials | not serious | not serious | not serious | not serious | none | 1019/1435 (71.0%) | 1058/1525 (69.4%) | **OR 1.06** (0.89 to 1.26) | **12 more per 1,000** (from 25 fewer to 47 more) | ⨁⨁⨁⨁ High | CRITICAL |
| **MRSA** | | | | | | | | | | | | |
| 5 | randomised trials | not serious | not serious | not serious | not serious | dose response gradient | 139/145 (95.9%) | 152/164 (92.7%) | **OR 1.29** (0.46 to 3.62) | **16 more per 1,000** (from 73 fewer to 52 more) | ⨁⨁⨁⨁ High | CRITICAL |
| **Microbiologic Response** | | | | | | | | | | | | |
| 6 | randomised trials | not serious | not serious | not serious | not serious | none | 779/855 (91.1%) | 798/902 (88.5%) | **OR 1.33** (0.94 to 1.88) | **26 more per 1,000** (from 6 fewer to 50 more) | ⨁⨁⨁⨁ High | CRITICAL |
| **MSSA** | | | | | | | | | | | | |
| 4 | randomised trials | not serious | not serious | not serious | not serious | none | 171/175 (97.7%) | 170/176 (96.6%) | **OR 1.38** (0.41 to 4.72) | **9 more per 1,000** (from 45 fewer to 27 more) | ⨁⨁⨁⨁ High | CRITICAL |
| **Staphylococcus aureus** | | | | | | | | | | | | |
| 5 | randomised trials | not serious | not serious | not serious | not serious | none | 308/318 (96.9%) | 321/340 (94.4%) | **OR 1.45** (0.65 to 3.23) | **17 more per 1,000** (from 28 fewer to 38 more) | ⨁⨁⨁⨁ High | CRITICAL |
|  | | | | | | | | | | | | |

**CI:** confidence interval; **OR:** odds ratio
